# Morphological and Molecular Characterization of a New Section and Two New Species of *Alternaria* from Iran

**DOI:** 10.3390/life15060870

**Published:** 2025-05-28

**Authors:** Abdollah Ahmadpour, Youbert Ghosta, Zahra Alavi, Fatemeh Alavi, Leila Mohammadi Hamidi, Pabulo Henrique Rampelotto

**Affiliations:** 1 Higher Education Center of Shahid Bakeri, Urmia University, Miyandoab 59781-59111, Iran; 2 Department of Plant Protection, Faculty of Agriculture, Urmia University, Urmia 57561-51818, Iran; y.ghoosta@urmia.ac.ir (Y.G.); alavizahra996@gmail.com (Z.A.); fatemeh.alavi0211@gmail.com (F.A.); leyla.m906@gmail.com (L.M.H.); 3 Bioinformatics and Biostatistics Core Facility, Institute of Basic Health Sciences, Federal University of Rio Grande do Sul, Porto Alegre 91501-970, Brazil

**Keywords:** *Alternaria*, multi-gene phylogeny, new section, new taxa, fungal biodiversity, taxonomy

## Abstract

*Alternaria* is a large genus of fungi comprising approximately 400 species, currently classified into 29 sections. These fungi exhibit a cosmopolitan distribution, thriving in both natural and human-impacted environments with saprophytic, endophytic, and parasitic lifestyles. As part of our ongoing studies on fungi associated with wetland plants in the families *Cyperaceae* and *Juncaceae* across various regions of Iran, we isolated 21 fungal strains displaying morphological traits of *Alternaria*. Multigene phylogenetic analysis and morphological examination of eight selected strains confirmed their placement within *Alternaria* with strong support. These isolates formed a basal clade distinct from the 29 previously recognized sections and six monotypic lineages, leading to the establishment of a new section, *Alternaria* section *Iraniana*, to accommodate them. Furthermore, two monophyletic lineages within this section were identified, representing two new species, *A. avrinica* and *A. iraniana*, which are described and illustrated in this study. The new section is distinguished by long, semi-macronematous to macronematous conidiophores with multiple geniculate and sympodial proliferations, as well as solitary, non-beaked conidia that have only transverse eu-septa to pseudo-septa. The newly described species are differentiated based on conidiophore and conidial characteristics and nucleotide sequence comparisons of genomic regions. These results contribute to a better understanding of the distribution and host range of *Alternaria* species, while highlighting the importance of ongoing research into fungal taxonomy and biodiversity in Iran, a region rich in potential for the discovery of new fungal species.

## 1. Introduction

The fungal genus *Alternaria* was established by Nees in 1816 on a single species, *A. tenuis* [1]. Nees originally described *Alternaria* as “erect, scattered, dark hyphae with separate three to four oval articulations, united by filiform connections”. However, this description lacked clarity and completeness, which complicated accurate species identification. Despite these limitations, Nees’s account is now considered sufficiently detailed to be recognized as referring to *Alternaria* [2,3]. In 1832, Fries introduced *Torula alternata* in *Systema Mycologicum*, without recognizing *Alternaria* as a distinct genus, and instead, listed *A. tenuis* Nees as a synonym of *T. alternata* [4]. Concurrently, Fries established the genus *Macrosporium*, which is morphologically similar to *Alternaria* [5]. The taxonomy of this group of phaeodictyosporic hyphomycetes became even more complex with the later descriptions of two additional genera, *Stemphylium* Wallroth (1833) and *Ulocladium* Preuss (1851) [6,7,8]. Von Keissler re-evaluated the descriptions of *A. tenuis* Nees and *T. alternata* Fr. and synonymized them under *A. alternata*, which is now recognized as the type species of *Alternaria* [9]. Although mycelium, conidiophores, and conidia served as diagnostic traits among these genera, differentiation primarily relied on conidial morphology. In an effort to revise the taxonomy and nomenclature of *Alternaria* and *Macrosporium*, the two genera were treated as separate, the generic concept of *Alternaria* was refined, and six morphological groups were proposed based on spore features [2]. The representative species of *Alternaria* and *Macrosporium* were later examined to clarify their classification [4]. It was concluded that *Alternaria* and *Macrosporium* are congeneric, and *Macrosporium* was discarded as a *nomen ambiguum* in favor of *Alternaria*.

Given the extensive morphological diversity within *Alternaria* and its related genera, efforts were made to classify the genus into subgeneric groups. Neergaard categorized *Alternaria* species in Denmark into three sections based on conidial catenulation, each typified by a species [10]. Species within each section were identified using conidial characteristics such as shape, size, color, surface ornamentation, and beak morphology. This classification led to the identification of 16 species, two varieties, and a few special forms. Similarly, Joly classified *Alternaria* species into three sections based on conidia color, rigidity, and lateral symmetry [11]. Simmons redefined the generic concepts of *Alternaria*, *Stemphylium*, and *Ulocladium* by examining authenticated specimens and emphasizing sporulation patterns, juvenile and mature conidial shapes, and modes of conidiophores and conidia proliferation [3]. *Alternaria alternata* (Fries) Keissler was formally designated as the type species of *Alternaria*. Ellis provided detailed descriptions and illustrations for 44 *Alternaria* taxa [12,13]. Subsequent studies expanded the number of species to approximately 70 [14,15,16,17,18,19]. Simmons introduced a species-group system based on conidial characteristics, the patterns of chain formation, and the nature of apical extensions of conidial cells, defining 14 species-groups [20]. Further species-groups were later described [21,22,23]. However, molecular phylogenetic studies reveal that many *Alternaria* species clades do not fully correspond with species-groups established based on morphology [8,22,23,24,25,26,27]. Lawrence et al. elevated species-groups to the taxonomic rank of section and established eight sections within the *Alternaria* complex, each typified by a type species [7]. However, the *A. infectoria* species-group was not granted section status. Woudenberg et al. redefined *Alternaria* using a combination of multi-gene phylogenetic analysis and morphological characteristics, emended its generic circumscription, added 16 new sections, identified six monotypic lineages, and synonymized 13 sexual and asexual generic names (*Allewia*, *Brachycladium*, *Chalastospora*, *Chmelia*, *Crivellia*, *Embellisia*, *Lewia*, *Nimbya*, *Sinomyces*, *Teretispora*, *Ulocladium*, *Undifilum*, and *Ybotromyces*) under *Alternaria* [8]. Subsequent studies added five more sections, bringing the total number of sections to 29 [28,29,30,31,32].

As part of our ongoing studies of fungi from various wetland plants in different regions of Iran, we isolated and purified 21 fungal strains with morphological characteristics similar to those of the genus *Alternaria*. Following a thorough morphological examination, eight strains were selected for further study using multi-gene phylogenetic analyses. The results indicate that these isolates represent a new basal section within the *Alternaria* complex, forming two monophyletic lineages and representing two new species. This study aims to introduce and describe the newly identified section, *Alternaria* section *Iraniana*, along with two new species: *Alternaria avrinica* and *A. iraniana*. Detailed morphological descriptions and illustrations are provided, along with a discussion of their phylogenetic relationships within the *Alternaria* complex.

## 2. Materials and Methods

### 2.1. Fungal Isolates

Leaf and culm samples exhibiting brown lesions and blight symptoms were collected from various wetland plants belonging to the families of *Cyperaceae* and *Juncaceae* across three provinces in Iran, Guilan, West Azarbaijan, and Zanjan, between 2019 and 2021. The samples were labeled, stored at low temperatures, and transported to the laboratory. Fungal isolation and purification followed the method described by Ahmadpour et al. [33]. The isolated fungal strains were preserved on Potato Dextrose Agar (PDA; 39 g/L, Merck, Darmstadt, Germany) slants at 4 °C and on sterile filter paper segments at –20 °C. All purified isolates were deposited in the fungal culture collections of the Iranian Research Institute of Plant Protection (IRAN) and Urmia University (FCCUU).

### 2.2. Morphological Characterization

For morphological characterization, purified isolates were cultured on Potato Carrot Agar (PCA) and incubated at 23–25 °C under Cool White fluorescent light, following an 8/16 h light/dark cycle for 5–7 days without humidity control [34,35]. Appropriate slide mounts were prepared using lactophenol, and microscopic features of the hyphae, conidiophores, and conidia were examined using an Olympus AX70 compound microscope with differential interference contrast (DIC) illumination (Olympus Optical CO., Ltd., Tokyo, Japan). Thirty to fifty structures of each type were measured, and microphotographs were captured from slide mounts and edited using Adobe Photoshop 2020 v. 2.10.8 software (Adobe Inc., San Jose, CA, USA). Colony morphology was assessed on PDA, PCA, and V-8A (comprising 175 mL of commercial V8 vegetable juice, 3 g CaCO_3_, 20 g agar, and 1000 mL distilled water) media after a 7-day incubation at 25 °C in the dark. Colony colors were identified using Rayner’s color charts [36]. Newly described taxa were registered in MycoBank [37].

### 2.3. DNA Extraction and PCR Amplification

Genomic DNA was extracted from fresh fungal mycelium scraped from 10-day-old PDA cultures using a standard sodium dodecyl sulfate (SDS) lysis buffer. The procedure included chloroform extraction and isopropanol precipitation [38]. Amplification targeted the small subunit rRNA (SSU), internal transcribed spacer (ITS-rDNA), large subunit rRNA (LSU), glyceraldehyde-3-phosphate dehydrogenase (*GAPDH*), RNA polymerase II largest subunit (*RPB2*), and translation elongation factor 1-alpha (*TEF1*) genes, using the primer pairs listed in Table 1. PCR conditions and reaction mixtures followed by Ahmadpour et al. [33,39]. Amplicons were analyzed on a 1% agarose gel stained with FluoroVue^TM^ Nucleic Acid Gel Stain (SMOBIO Technology Inc., Hsinchu, Taiwan), and fragment sizes were compared against a FluoroBand^TM^ 100 bp+3K Fluorescent DNA Ladder (SMOBIO Technology Inc., Hsinchu, Taiwan). PCR products were cleaned and sequenced by Macrogen Corp. (Seoul, South Korea) using the same primers as in PCR amplification. The resulting sequences were submitted to GenBank (Table 2).

### 2.4. Sequence Alignment and Phylogenetic Analyses

The generated sequences were processed and trimmed using MEGA 6.0 before being exported as FASTA files for further analysis [45]. Corresponding sequences from type or representative *Alternaria* strains were retrieved from the GenBank database and incorporated into phylogenetic analyses (Table 2) [7,8,23,27,28,29,30,31,32,36,46,47]. Multiple sequence alignments for each locus were performed using the MAFFT version 7 online tool (https://mafft.cbrc.jp/alignment/server/; accessed on 20 February 2025) [48], with additional manual refinements in MEGA 6.0 as needed. Concatenated multi-gene datasets were assembled using Mesquite v. 3.61 [49]. Two separate multi-locus phylogenetic analyses were conducted: (1) incorporating 167 representative strains from five families in the suborder *Pleosporineae*, using five gene sequences (SSU, ITS, LSU, *GAPDH*, and *RPB2*) to determine the placement of the studied isolates, and (2) incorporating 110 representative strains from 29 *Alternaria* sections and six monotypic lineages, using six gene sequences (SSU, ITS, LSU, *GAPDH*, *RPB2*, and *TEF1*), to resolve the placement of strains within the *Alternaria* complex. Bayesian inference (BI) analysis was performed in MrBayes v. 3.2.7 [50] using the Markov Chain Monte Carlo method with four chains, one-million generations, and a heated chain temperature of 0.1. Trees were sampled every 1000 generations, with a 25% burn-in, and posterior probabilities were calculated from the remaining trees. Convergence was confirmed when the average standard deviation of split frequencies dropped below 0.01. The best-fit evolutionary models for BI were determined using MrModeltest 2.3 [51] and the Akaike Information Criterion (AIC) (Table 3). Maximum-likelihood (ML) analysis was conducted using RAxML-HPC BlackBox v. 8.2.12 [52] via the CIPRES Science Gateway version 3.3 (accessible at https://www.phylo.org/; accessed on 20 February 2025) [53], applying the GTRGAMMA+I substitution model. Maximum Parsimony (MP) analysis was performed in PAUP v. 4.0b10 [54], using a heuristic search with 1000 random sequence additions and tree-bisection-reconnection (TBR) branch swapping while treating gaps as missing data. Bootstrap support values were estimated from 1000 replicates, and tree statistics—including Tree Length (TL), Consistency Index (CI), Retention Index (RI), and Homoplasy Index (HI)—were calculated for the MP analysis. Outgroup taxa consisted of *Halojulella avicenniae* (BCC 18422) for the first analysis, and *Stemphylium botryosum* (CBS 714.68) and *S. vesicarium* (CBS 191.86) for the second analysis. The final phylogenetic trees were visualized using FigTree v. 1.4.4 [55].

## 3. Results

### 3.1. Phylogenetic Analyses

**Figure 1:**
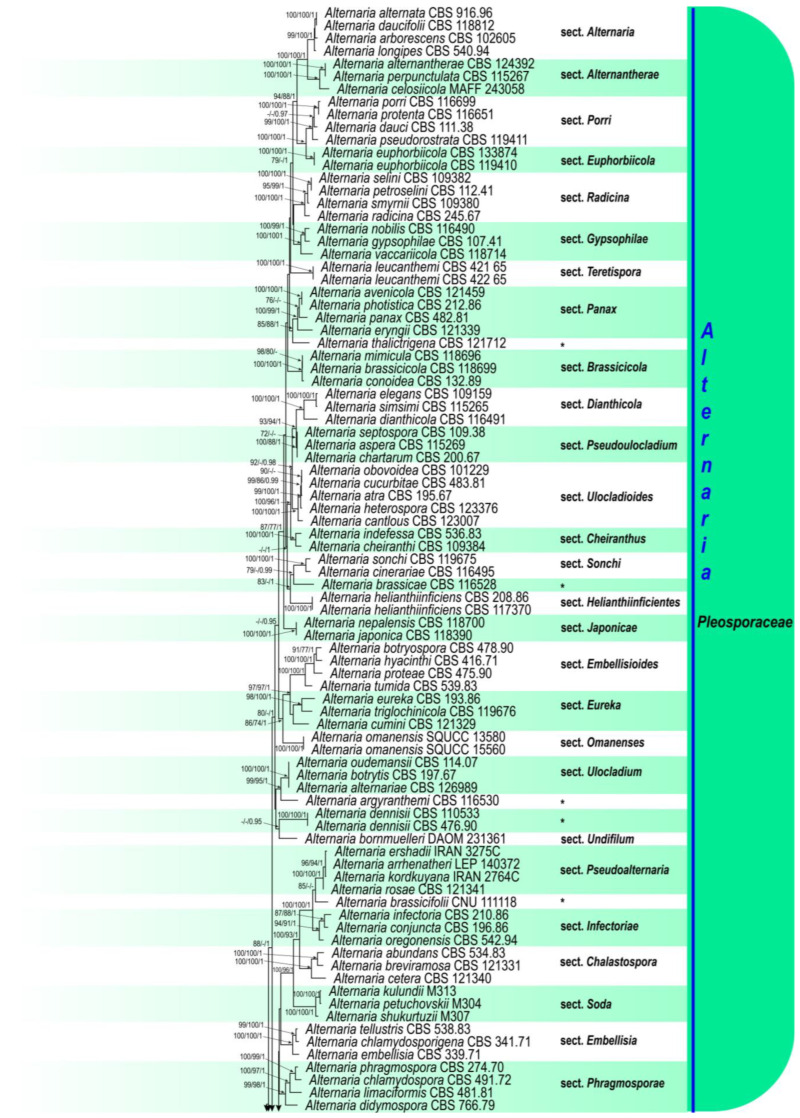

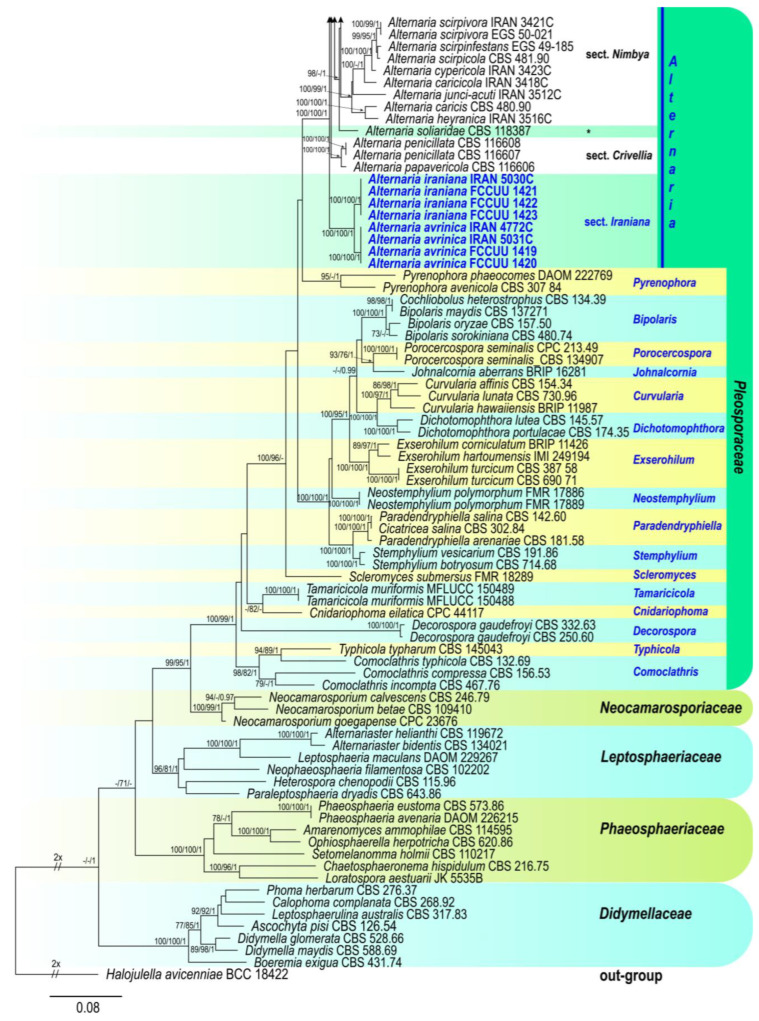
Phylogenetic tree generated using maximum-likelihood (ML) analysis of 167 strains in the suborder *Pleosporineae*, based on a concatenated dataset of SSU, ITS, LSU, *GAPDH*, and *RPB2* sequences. Bootstrap support values for maximum likelihood and maximum parsimony (MLBS/MPBS) ≥70% and Bayesian posterior probabilities (BIPP) ≥ 0.95 are shown at the nodes. The tree is rooted with *Halojulella avicenniae* (BCC 18422). The scale bar represents the number of nucleotide substitutions. Newly identified strains are highlighted in bold blue. Families, genera, and sections are presented with colored blocks and * indicate monotypic lineages.

**Figure 2:**
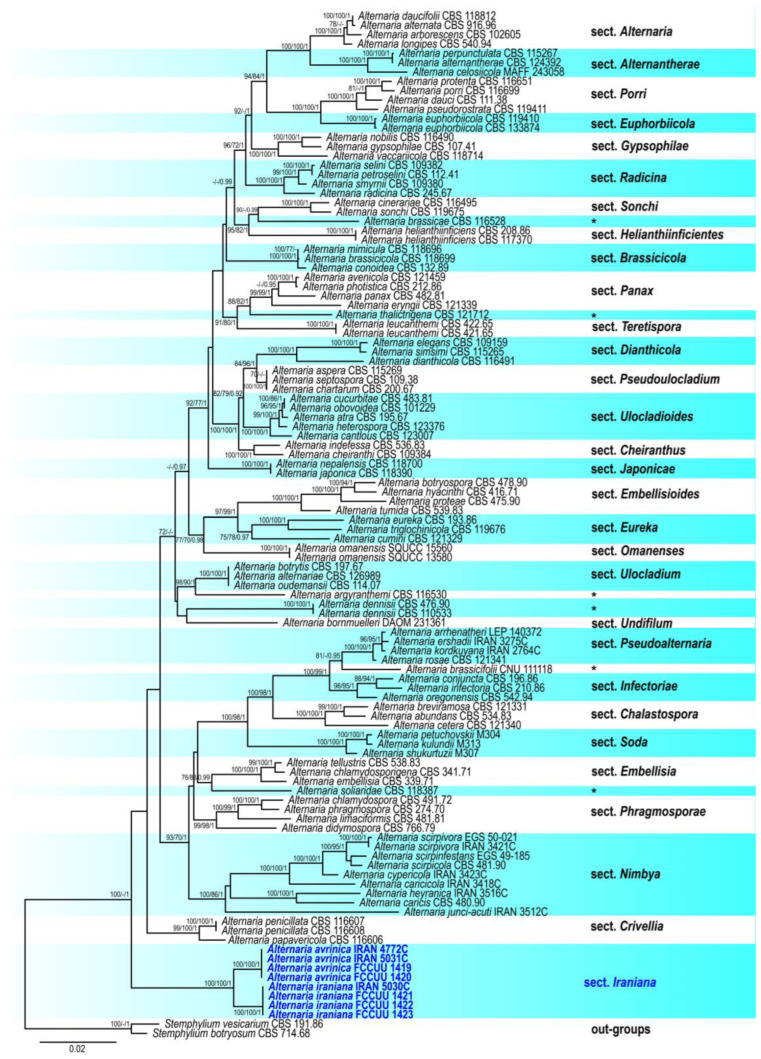
Phylogenetic tree generated using maximum-likelihood (ML) analysis of 110 *Alternaria* spp. strains based on a concatenated dataset of SSU, ITS, LSU, *GAPDH*, *RPB2*, and *TEF1* sequences. Bootstrap support values for maximum likelihood and maximum parsimony (MLBS/MPBS) ≥ 70% and Bayesian posterior probabilities (BIPP) ≥ 0.95 are shown at the nodes. The tree is rooted with *Stemphylium botryosum* (CBS 714.68) and *S. vesicarium* (CBS 191.86). Newly identified strains are highlighted in bold blue. The scale bar represents the number of nucleotide substitutions. Six monotypic lineages are marked with a black asterisk.

The results of the first phylogenetic analysis, which included 167 representative strains from five families in the suborder *Pleosporineae*, are presented in Figure 1. The eight studied strains were placed within the large *Alternaria* clade with strong support (ML/MP/BI = 100/100/1), confirming their identities as *Alternaria*. The concatenated matrix, which used SSU, ITS, LSU, *GAPDH*, and *RPB2* sequences, contained a total of 4050 characters, including gaps (1373 SSU, 437 for ITS, 689 LSU, 534 for *GAPDH*, and 837 for *RPB2*). Of these, 2807 were constant sites, 1243 were variable sites, 292 were parsimony-uninformative sites, and 951 were parsimony-informative sites (Table 3). The most parsimonious tree yielded the following metrics: TL = 8516, CI = 0.241, RI = 0.686, HI = 0.759. The results of the second phylogenetic analysis, which included 110 representative strains from 29 *Alternaria* sections and 6 monotypic lineages, are presented in Figure 2. The studied isolates formed a distinct subclade at the base of the large *Alternaria* clade and comprised two monophyletic lineages, representing two different species. The concatenated matrix, which used SSU, ITS, LSU, *GAPDH*, *RPB2*, and *TEF1* sequences, contained a total of 4003 characters including gaps (1022 for SSU, 457 for ITS, 851 for LSU, 557 for *GAPDH*, 799 for *RPB2*, and 317 for *TEF1*). Of these, 3105 were constant sites, 898 were variable sites, 121 were parsimony-uninformative sites, and 777 were parsimony-informative sites (Table 3). The most parsimonious tree yielded the following metrics: TL = 4299, CI = 0.329, RI = 0.728, HI = 0.671. The topologies of individual gene trees were consistent, with no conflicts observed in species delimitation. The best-scoring RAxML trees are shown in Figure 1 and Figure 2. A summary of phylogenetic information and substitution models for each dataset is provided in Table 3. Based on the phylogenetic results and morphological characteristics, a new section, *Alternaria* section *Iraniana*, and two new species, *A. avrinica* and *A. iraniana* (the type species of the new section), are introduced and described.

### 3.2. Taxonomy

A total of 21 fungal isolates were obtained from various plants in the families *Cyperaceae* and *Juncaceae*. All isolates were examined morphologically, and eight representative isolates from different plant hosts were selected for phylogenetic analyses. Based on phylogenetic and morphological analyses, the studied isolates were assigned to a new section, designated here as *Alternaria* section *Iraniana*, which includes two new species: *A. avrinica* and *A. iraniana*. Detailed morphological descriptions and illustrations of the new taxa are provided, along with a discussion of their phylogenetic relationships with other species in the *Alternaria* sections.

#### 3.2.1. Section *Iraniana* A. Ahmadpour, Y. Ghosta, Z. Alavi, F. Alavi & L. Mohammadi, sect. nov.

MycoBank No. MB 858618

Type species. *Alternaria iraniana* A. Ahmadpour, Y. Ghosta, Z. Alavi, F. Alavi & L. Mohammadi

Diagnosis. Members of *Alternaria* section *Iraniana* are characterized by long, simple, semi-macronematous to macronematous conidiophores with multiple geniculate, sympodial proliferation and mono- to polytretic conidiogenous loci. Conidia are solitary, ellipsoidal to cylindrical, non-beaked, and contain only transverse eu- or pseudo-septa. Apical cells in some conidia germinate with the production of a secondary conidiophore, bearing solitary conidia, while they are attached to primary conidiophores. Sexual morph was not observed. This section is associated with plants in the families *Cyperaceae* and *Juncaceae*.

Notes. Based on phylogenetic analyses, the studied isolates formed a well-separated basal clade within the large *Alternaria* complex, distinct from all other known sections and monotypic lineages. Section *Crivellia* is the closest relative to the section *Iraniana* (Figure 1 and Figure 2).

#### 3.2.2. *Alternaria avrinica* A. Ahmadpour, Y. Ghosta, Z. Alavi, F. Alavi & L. Mohammadi, sp. nov. (Figure 3)

MycoBank No. MB 858619

Etymology. The name refers to Avrin Mountain, located in Khoy County, West Azarbaijan province, where the holotype was collected.

Diagnosis: Differs from *A. iraniana* by the size of primary conidiophores, conidia, and absence of secondary conidiophores.

Typification. Iran, West Azarbaijan province, Khoy County, Avrin Mountain, isolated from the culms of *Juncus* sp. (*Juncaceae*, *Poales*), 20 September 2021, *A. Ahmadpour* (holotype IRAN 18204F, ex-type culture IRAN 4772C).

Description. *Asexual morph* on PCA medium: *Hyphae* 2–4 μm wide, pale brown to brown, smooth, septate, branched. *Conidiophores* (32–)47–187(–250) × 4–5 µm ($\bar{x}$ = 118 × 4.5 μm, *n* = 50), mononematous, semi-macronematous to macronematous, unbranched, straight to flexuous, septate, geniculate, brown to dark brown, paler towards the apex, rarely swollen at the base. *Conidiogenous cells* mono- to polytretic, sympodial proliferation, integrated, terminal or intercalary, subcylindrical to slightly swollen, pale brown to brown, smooth-walled, with thickened and darkened scars. *Conidia* (9–)12–30(–35) × 5–7 µm ($\bar{x}$ = 23 × 6 μm, *n* = 50), solitary, pale brown to brown, smooth-walled, straight, ellipsoidal to cylindrical, tapering towards rounded ends, without beak, 1–5 transverse septate, (of which 1–4 (mostly 3) are eu-septate and 1–2 disto-septate), without longitudinal and oblique septa; hila 1–2 μm wide, inconspicuous, flat, thickened, and darkened. *Sexual morph* and *chlamydospores* were not observed.

**Figure 3:**
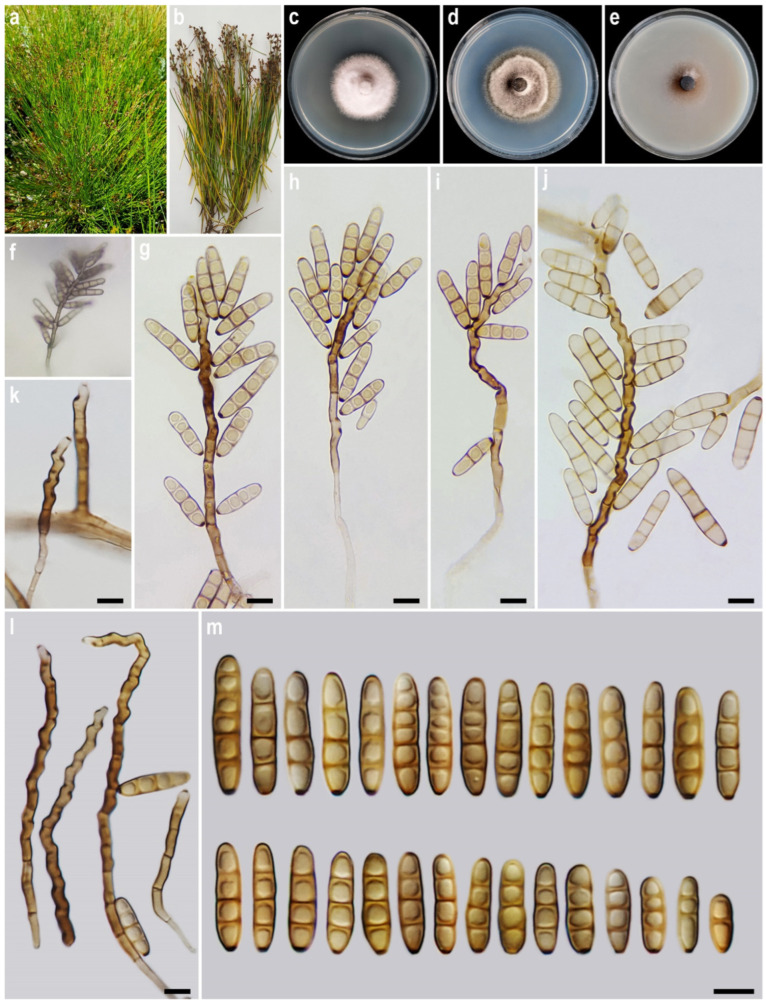
*Alternaria avrinica* (IRAN 4772C). (**a**,**b**) Host (*Juncus* sp.); (**c**–**e**) Colony on PDA (**c**), PCA (**d**), and V-8A (**e**) after 7 days; (**f**) Sporulation pattern on PCA (40×); (**g**–**m**) Conidiophores and conidia. Scale bars: (**g**–**m**) = 10 μm.

Culture characteristics. Colony on PCA 42 mm diam., after 7 days at 25 °C, flat, entire, circular, margin regular, olivaceous grey, with white to grey aerial mycelia; reverse olivaceous grey. Colony on PDA 39 mm diam., flat, entire, circular, margin regular, cottony appearance, with white to grey aerial mycelia; reverse grey. Colony on V-8A 26 mm diam., flat, entire, circular, margin entire, hairy appearance, grey, with sparse white to grey aerial mycelia; reverse grey at the center and hyaline at the margin. Sporulation abundant on PCA, and V-8A media, from the erect conidiophores that arise directly from the surface or the aerial hyphae.

Additional specimens examined. Iran, West Azarbaijan province, Khoy County, Avrin Mountain, on infected culms of *Juncus* sp., 20 September 2021, *A. Ahmadpour* (culture IRAN 5031C). Iran, Zanjan province, Tarom County, Gilvan city, on infected leaves and culms of *Carex* sp. (*Cyperaceae*, *Poales*), 11 September 2022, *A. Ahmadpour* (culture FCCUU 1419). —*ibid.* on infected culms of *Juncus* sp., 11 September 2022, *A. Ahmadpour* (culture FCCUU 1420).

Notes. *Alternaria avrinica* is morphologically similar and phylogenetically closely related to *A. iraniana*, *A. papavericola*, and *A. penicillata*. This species can be differentiated from *A. iraniana* by its larger conidia (9–35 × 5–7 vs. 10–32 × 4–6 μm), shorter primary conidiophores (32–250 vs. 50–337 μm), and the absence of secondary conidiophores; from *A. papavericola* by longer conidiophores (32–250 vs. 20–130 μm), smaller conidia (9–35 × 5–7 vs. 40–70 × 6–8 μm), fewer transverse septa (1–5 vs. 3–8), and absence of chlamydospores and ascomata (melanized chlamydospore-like thick-walled cells are commonly formed in *A. papavericola*); and from *A. penicillata* by longer conidiophores (32–250 vs. 30–60 μm), and the absence of microsclerotia and ascomata [56]. A comparison of nucleotide differences in SSU, ITS, LSU, *GAPDH*, *RPB2*, and *TEF1* indicates that *A. avrinica* type strain (IRAN 4772C) differs from *A. iraniana* type strain (IRAN 5030C) by 6/513 bp (1.16%, with 3 gaps (0%)) in ITS, 3/810 bp (0.37%) in LSU, 13/567 bp (2.29%, with 4 gaps (0%)) in *GAPDH*, 28/762 bp (3.67%) in *RPB2* and 5/183 bp (2.73%) in *TEF1*, from *A. papavericola* type strain (CBS 116606) by 2/807 bp (0.24%, with one gap (0%)) in SSU, 27/515 bp (5.24%, with 8 gaps (1%)) in ITS, 3/833 bp (0.36%) in LSU, 61/557 bp (10.95%, with 10 gaps (1%)) in *GAPDH*, 63/793 bp (7.94%) in *RPB2* and 23/179 bp (12.84%, with 3 gaps (1%)) in *TEF1* and from *A. penicillata* type strain (CBS 116608) by 2/807 bp (0.24%, with one gap (0%)) in SSU, 32/515 bp (6.21%, with 7 gaps (1%)) in ITS, 3/833 bp (0.36%) in LSU, 65/559 bp (11.62%, with 16 gaps (2%)) in *GAPDH*, 68/793 bp (8.57%) in *RPB2* and 17/179 bp (9.49%, with 3 gaps (1%)) in *TEF1*.

#### 3.2.3. *Alternaria iraniana* A. Ahmadpour, Y. Ghosta, Z. Alavi, F. Alavi & L. Mohammadi, sp. nov. (Figure 4)

MycoBank No: MB 858620

Etymology. Named after the country “Iran”, where the holotype was collected.

Typification. Iran, Guilan province, Asalem County, isolated from the culms of *Juncus* sp. (*Juncaceae*, *Poales*), 9 September 2022, *A. Ahmadpour* (holotype IRAN 18473F, ex-type culture IRAN 5030C).

Description. *Asexual morph* on PCA medium: *Hyphae* 2–4 μm wide, pale brown to brown, smooth, septate, branched. *Conidiophores* (50–)75–250(–337) × 4–5 µm ($\bar{x}$ = 150 × 4.5 μm, *n* = 50), mononematous, semi-macronematous to macronematous, unbranched, straight to flexuous, septate, geniculate, brown to dark brown, paler towards the apex. *Conidiogenous cells* mono- to polytretic, sympodial proliferation, integrated, terminal or intercalary, subcylindrical to slightly swollen, pale brown to brown, smooth-walled, with thickened and darkened scars. Apical cells in some conidia germinate with the production of a secondary conidiophore, resembling primary conidiophores in size and geniculations, bearing solitary conidia, while they are attached to primary conidiophores. *Conidia* (10–)13–29(–32) × 4–6 µm ($\bar{x}$ = 20 × 5 μm, *n* = 50), solitary, pale brown to brown, smooth-walled, straight, ellipsoidal to cylindrical, 1–4 transverse septa, (of which 1–3 (mostly 3) are eu-septate and 1–3 disto-septate), without longitudinal and oblique septa; hila 1–2 μm wide, inconspicuous, flat, thickened, and darkened. *Sexual morph* and *chlamydospores* were not observed.

**Figure 4:**
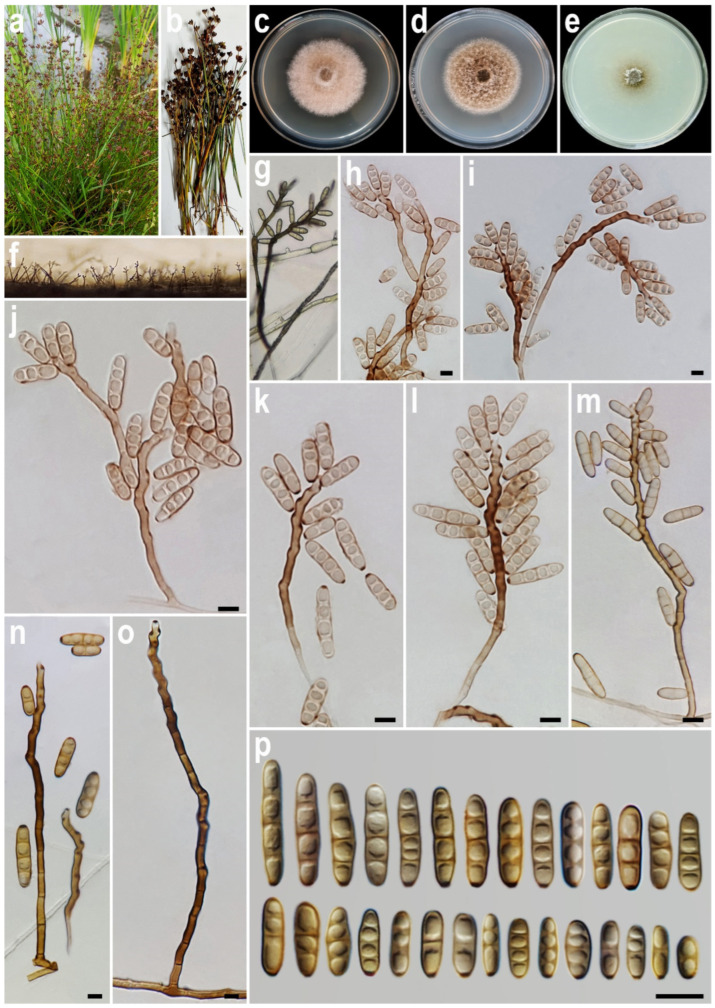
*Alternaria iraniana* (IRAN 5030C). (**a**,**b**) Host (*Juncus* sp.); (**c**–**e**) Colony on PDA (**c**), PCA (**d**), and V-8A (**e**) after 7 days; (**f**,**g**) Sporulation pattern on PCA (f = 10×, g = 40×); (**h**–**p**) Conidiophores and conidia. Scale bars: (**h**–**p**) = 10 μm.

Culture characteristics. Colony on PCA 50 mm diam., after 7 days at 25 °C, flat, entire, circular, margin regular, grey with white to grey aerial mycelia; reverse grey. Colony on PDA 46 mm diam., flat, entire, circular, margin regular, velvety, with white to grey aerial mycelia; reverse pale brown at the center to grey at the margin. Colony on V-8A 22 mm diam., flat, entire, circular, margin entire, hairy appearance, grey with sparse white to grey aerial mycelia; reverse grey at the center and hyaline at the margin. Sporulation abundant on PCA, and V-8A media, from the erect conidiophores that arise directly from the surface or the aerial hyphae.

Additional specimens examined. Iran, Guilan province, Asalem County, isolated from the culms of *Juncus* sp. (*Juncaceae*, *Poales*), 9 September 2022, *A. Ahmadpour* (cultures FCCUU 1421, FCCUU 1422, FCCUU 1423).

Notes. *Alternaria iraniana* is morphologically similar and phylogenetically closely related to *A. avrinica*, *A. papavericola*, and *A. penicillata*. This species can be differentiated from *A. avrinica* by its smaller conidia (10–32 × 4–6 vs. 9–35 × 5–7 μm), longer primary conidiophores (50–337 vs. 32–250 μm), and the presence of secondary conidiophores; from *A. papavericola* by longer conidiophores (50–337 vs. 20–130 μm), smaller conidia (10–32 × 4–6 vs. 40–70 × 6–8 μm), fewer transverse septa (1–4 vs. 3–8); and from *A. penicillata* by longer conidiophores (50–337 vs. 30–60 μm), smaller conidia (10–32 × 4–6 vs. 17–35 × 5–7) and the absence of microsclerotia and ascomata [56]. Comparisons of nucleotide differences in *A. iraniana* are provided in the notes section for *A. avrinica*. A comparison of nucleotide differences in ITS, LSU, *GAPDH*, *RPB2*, and *TEF1* indicates that *A. iraniana* type strain (IRAN 5030C) differs from *A. papavericola* type strain (CBS 116606) by 29/545 bp (5.32%, with 6 gaps (1%)) in ITS, 2/826 bp (0.24%) in LSU, 66/560 bp (11.78%, with 14 gaps (2%)) in *GAPDH*, 64/829 bp (7.72%, with 1 gaps (0%)) in *RPB2* and 33/221 bp (14.93%, with 5 gaps (2%)) in *TEF1* and from *A. penicillata* type strain (CBS 116608) by 31/515 bp (5.67%, with 6 gaps (1%)) in ITS, 2/826 bp (0.14%) in LSU, 59/558 bp (10.57%, with 8 gaps (1%)) in *GAPDH*, 68/829 bp (8.20%, with 1 gaps (0%)) in *RPB2* and 27/221 bp (12.21%, with 5 gaps (2%)) in *TEF1*.

## 4. Discussion

*Alternaria* (*Pleosporaceae*, *Pleosporales*, *Dothideomycetes*, *Ascomycota*) is a widely distributed fungal genus found in diverse habitats and substrates, including agricultural products, animals, the atmosphere, food and feed commodities, indoor environments, plants, and soil [8,57,58,59]. According to the Index Fungorum database (https://www.indexfungorum.org/; accessed 29 March 2025), 861 species epithets have been recorded, although only approximately 400 species have been formally described [33,39,60,61,62,63,64,65]. Many species in this genus are plant pathogens, responsible for destructive diseases in more than 400 plant species, and some act as post-harvest pathogens, contributing to losses of horticultural products [66,67,68,69]. Additionally, some *Alternaria* species are opportunistic pathogens in animals and humans or serve as airborne allergens. They are also well-known for producing toxic secondary metabolites, including mycotoxins and host-specific toxins, which pose serious food safety risks when contaminating food and feed [70,71,72,73,74,75,76,77]. Accurate species identification is essential in both fundamental and applied fungal research, aiding in the understanding of fungal biology, biodiversity, evolution, and ecological impact [56,57,58,59,60,61,62,63,64,65,66,67,68,69,70,71,72,73,74,75,76,77,78,79,80,81]. Traditionally, species identification has relied on morphological characteristics of asexual/sexual morphs, with host relationships being a secondary factor [20,35,82,83,84]. However, phylogenetic studies demonstrate that these morphological classifications do not always reflect true evolutionary relationships [28,46,85]. Currently, species delimitation is based on multi-locus sequence analysis combined with morphological characteristics; however, various gene combinations are used for species identification, depending on the specific sections of *Alternaria* [8,32,69,86,87]. These advances have refined *Alternaria* taxonomy, clarified species boundaries, and facilitated the regular description of new species.

*Cyperaceae* and *Juncaceae* are two families of graminoid, monocotyledonous flowering plants that dominate wetland ecosystems. They provide essential ecological functions, such as habitat and food for wildlife, nutrient cycling, and water purification. In addition, they have various traditional applications in cosmetics, medicine, perfumery, and handicrafts. However, they are also considered invasive weeds that compete with crop plants and harbor diverse fungal communities [88,89,90,91,92,93,94]. Identifying and characterizing fungi associated with these plants is of primary relevance for understanding their impact on host health, host range, and potential for disease management, biocontrol applications, and biotechnology. In our study on fungi associated with plants in families *Cyperaceae* and *Juncaceae*, 21 strains resembling the genus *Alternaria* were isolated and purified. These strains exhibited simple, elongated, geniculately proliferating conidiophores, and solitary beakless conidia with only transverse septa, both eu-septa and distosepta. The morphological characteristics of conidiophores, especially successive geniculations with porospores and the formation of solitary conidia at each condiogenous locus, are somewhat similar to those of *Pleosporaceae* genera (*Bipolaris*, *Curvularia*, *Exserohilum*, *Johnalcornia*, and *Pyrenophora*). However, the conidial shape and the formation of transverse eu-septa distinguish the new species from those of other *Pleosporaceae* genera [95,96,97]. Multi-gene phylogenetic analysis confirmed that the studied strains form a well-supported monophyletic clade within *Alternaria* (Figure 1 and Figure 2), positioned as a basal clade relative to other *Alternaria* sections and monotypic lineages. Based on these findings, we propose a new section, *Iraniana*. The topology of our phylogenetic tree aligns with those of Lawrence et al. [8] and Li et al. [59]. The closest related section, *Crivellia*, includes two species, *A. penicillata*, and *A. papavericola*, isolated from *Papaver* spp. (*Papaveraceae*) [56]. While species in both sections share similar conidial morphology and septation patterns, members of section *Iraniana* lack microsclerotia/chlamydospore formation and sexual morphs (pseudothecia), which are observed in section *Crivellia*. Within section *Iraniana*, two species were identified and differentiated based on morphological characteristics and nucleotide sequence comparisons. In our previous studies, we identified 13 species of *Alternaria* within section *Nimbya* [33,38,39]. The present study further highlights that plants in the families of *Cyperaceae* and *Juncaceae* serve as primary hosts of previously undescribed *Alternaria* species. Additional research is required to explore their full diversity in wetlands, assess the pathogenicity and host range of these newly identified strains, and evaluate their potential for biocontrol of weedy wetland plants. On fostering interdisciplinary collaboration, we can better address the challenges posed by these fungi and leverage their potential benefits in sustainable agriculture and ecosystem management.

## Figures and Tables

**Table 1:** Primer sets used for PCR amplifications in this study, their sequences, and references.

Loci	Primer Name	Primer Sequence (5′–3′)	Direction	References
SSU	NS1	GTAGTCATATGCTTGTCTC	Forward	[40]
	NS4	CTTCCGTCAATTCCTTTAAG	Reverse	
ITS	ITS1	TCCGTAGGTGAACCTGCGG	Forward	[40]
	ITS4	TCCTCCGCTTATTGATATGC	Reverse	
LSU	LR0R	GTACCCGCTGAACTTAAGC	Forward	[40]
	LR5	TCCTGAGGGAAACTTCG	Reverse	
*GAPDH*	gpd1	CAACGGCTTCGGTCGCATTG	Forward	[41]
	gpd2	GCCAAGCAGTTGGTTGTG	Reverse	
*RPB2*	RPB2-5F2	GGGGWGAYCAGAAGAAGGC	Forward	[42]
	RPB2-7cR	CCCATRGCTTGTYYRCCCAT	Reverse	[43]
*TEF1*	EF1-728F	CATCGAGAAGTTCGAGAAGG	Forward	[44]
	EF1-986R	TACTTGAAGGAACCCTTACC	Reverse	

**Table 2:** Lists of the *Alternaria* species and allied genera in *Pleosporineae* used for phylogenetic analyses, with details about host/substrate, country, and GenBank accession numbers. Newly generated sequences are in bold. ^R^ and ^T^ indicate reference and ex-type strains, respectively.

Species Name	Section	Collection No.	Country	Host/Substrate	GenBank Accession Numbers					
					SSU	ITS	LSU	*GAPDH*	*RPB2*	*TEF1*
*Alternaria abundans*	*Chalastospora*	CBS 534.83 ^T^	New Zealand	*Fragaria* sp.	KC584581	JN383485	KC584323	KC584154	KC584448	KC584707
*A. alternantherae*	*Alternantherae*	CBS 1243s92	China	*Solanum melongena*	KC584506	KC584179	KC584251	KC584096	KC584374	KC584633
*A. alternariae*	*Ulocladium*	CBS 126989 ^T^	USA	*Daucus carota*	KC584604	AF229485	KC584346	AY278815	KC584470	KC584730
*A. alternata*	*Alternaria*	CBS 916.96 ^T^	India	*Arachis hypogaea*	KC584507	AF347031	DQ678082	AY278808	KC584375	KC584634
*A. arborescens*	*Alternaria*	CBS 102605 ^T^	USA	*Lycopersicon* *esculentum*	KC584509	AF347033	KC584253	AY278810	KC584377	KC584636
*A. argyranthemi*	-	CBS 116530 ^T^	New Zealand	*Argyranthemum* sp.	KC584510	KC584181	KC584254	KC584098	KC584378	KC584637
*A. arrhenatheri*	*Pseudoalternaria*	LEP 140372 ^T^	USA	*Arrhenatherum elatius*	-	JQ693677	-	JQ693635	-	-
*A. aspera*	*Pseudoulocladium*	CBS 115269 ^T^	Japan	*Pistacia vera*	KC584607	KC584242	KC584349	KC584166	KC584474	KC584734
*A. atra*	*Ulocladioides*	CBS 195.67 ^T^	USA	Soil	KC584608	AF229486	KC584350	KC584167	KC584475	KC584735
*A. avenicola*	*Panax*	CBS 121459 ^T^	Norway	*Avena* sp.	KC584512	KC584183	KC584256	KC584100	KC584380	KC584639
***A. avrinica***	***Iraniana***	**IRAN 4772C ^T^**	**Iran**	***Juncus* sp.**	**PV435164**	**PV435148**	**PV435156**	**PV443215**	**PV443231**	**PV443223**
***A. avrinica***	***Iraniana***	**IRAN 5031C**	**Iran**	***Juncus* sp.**	**PV435165**	**PV435149**	**PV435157**	**PV443216**	**PV443232**	**PV443224**
***A. avrinica***	***Iraniana***	**FCCUU 1419**	**Iran**	***Carex* sp.**	**PV435166**	**PV435150**	**PV435158**	**PV443217**	**PV443233**	**PV443225**
***A. avrinica***	***Iraniana***	**FCCUU 1420**	**Iran**	***Juncus* sp.**	**PV435167**	**PV435151**	**PV435159**	**PV443218**	**PV443234**	**PV443226**
*A. bornmuelleri*	*Undifilum*	DAOM 231361 ^T^	Austria	*Securigera varia*	KC584624	FJ357317	KC584366	FJ357305	KC584491	KC584751
*A. botryospora*	*Embellisioides*	CBS 478.90 ^T^	New Zealand	*Leptinella dioica*	KC584594	MH862228	KC584336	AY278831	KC584461	KC584720
*A. botrytis*	*Ulocladium*	CBS 197.67 ^T^	USA	Contaminant	KC584609	KC584243	KC584351	KC584168	KC584476	KC584736
*A. brassicae*	-	CBS 116528 ^R^	USA	*Brassica oleracea*	KC584514	KC584185	KC584258	KC584102	KC584382	KC584641
*A. brassicicola*	*Brassicicola*	CBS 118699 ^R^	USA	*Brassica oleracea*	KC584515	JX499031	KC584259	KC584103	KC584383	KC584642
*A. brassicifolii*	-	CNU 111118 ^T^	Korea	*Brassica rapa* subsp. *pekinensis*	-	JQ317188	-	KM821537	-	-
*A. breviramosa*	*Chalastospora*	CBS 121331 ^T^	Australia	*Triticum* sp.	KC584574	FJ839608	KC584318	KC584148	KC584442	KC584700
*A. cantlous*	*Ulocladioides*	CBS 123007 ^T^	China	*Cucumis melo*	KC584612	KC584245	KC584354	KC584171	KC584479	KC584739
*A. caricicola*	*Nimbya*	IRAN 3418C ^T^	Iran	*Carex* sp.	-	MK508871	-	MK505392	MT187279	MT187265
*A. caricis*	*Nimbya*	CBS 480.90 ^T^	USA	*Carex hoodii*	KC584600	AY278839	KC584342	AY278826	KC584467	KC584726
*A. celosiicola*	*Alternantherae*	MAFF 243058	Japan	*Celosia argentea* var. *plumosa*	-	AB678217	-	AB744033	LC476781	LC480205
*A. cetera*	*Chalastospora*	CBS 121340 ^T^	Australia	*Elymus scabrus*	KC584573	JN383482	KC584317	AY562398	KC584441	KC584699
*A. chartarum*	*Pseudoulocladium*	CBS 200.67 ^T^	Canada	*Populus* sp.	KC584614	AF229488	KC584356	KC584172	KC584481	KC584741
*A. cheiranthi*	*Cheiranthus*	CBS 109384^R^	Italy	*Cheiranthus cheiri*	KC584519	AF229457	KC584263	KC584107	KC584387	KC584646
*A. chlamydospora*	*Phragmosporae*	CBS 491.72 ^T^	Egypt	Soil	KC584520	KC584189	KC584264	KC584108	KC584388	KC584647
*A. chlamydosporigena*	*Embellisia*	CBS 341.71 ^R^	USA	Air	KC584584	KC584231	KC584326	KC584156	KC584451	KC584710
*A. cinerariae*	*Sonchi*	CBS 116495 ^R^	USA	*Ligularia* sp.	KC584521	KC584190	KC584265	KC584109	KC584389	KC584648
*A. conjuncta*	*Infectoriae*	CBS 196.86 ^T^	Switzerland	*Pastinaca sativa*	KC584522	FJ266475	KC584266	AY562401	KC584390	KC584649
*A. conoidea*	*Brassicicola*	CBS 132.89	Saudi Arabia	*Ricinus communis*	KC584585	FJ348226	KC584327	FJ348227	KC584452	KC584711
*A. cucurbitae*	*Ulocladioides*	CBS 483.81^R^	New Zealand	*Cucumis sativus*	KC584616	FJ266483	KC584358	AY562418	KC584483	KC584743
*A. cumini*	*Eureka*	CBS 121329 ^T^	India	*Cuminum cyminum*	KC584523	KC584191	KC584267	KC584110	KC584391	KC584650
*A. cypericola*	*Nimbya*	IRAN 3423C ^T^	Iran	*Cyperus* sp.	-	MT176120	-	MT187250	MT187276	MT187262
*A. dauci*	*Porri*	CBS 111.38 ^T^	Italy	*Daucus carota*	-	KJ718158	-	KJ718005	KJ718331	KJ718506
*A. daucifolii*	*Alternaria*	CBS 118812 ^T^	USA	*Daucus carota*	KC584525	KC584193	KC584269	KC584112	KC584393	KC584652
*A. dennisii*	-	CBS 476.90 ^T^	Isle of Man	*Senecio jacobaea*	KC584587	JN383488	KC584329	JN383469	KC584454	KC584713
*A. dennisii*	-	CBS 110533	New Zealand	*Senecio jacobaea*	KC584586	KC584232	KC584328	KC584157	KC584453	KC584712
*A. dianthicola*	*Dianthicola*	CBS 116491 ^R^	New Zealand	*Dianthus* × *allwoodii*	KC584526	KC584194	KC584270	KC584113	KC584394	KC584653
*A. didymospora*	*Phragmosporae*	CBS 766.79	Adriatic Sea	Seawater	KC584588	FJ357312	KC584330	FJ357300	KC584455	KC584714
*A. elegans*	*Dianthicola*	CBS 109159 ^T^	Burkina Faso	*Lycopersicon esculentum*	KC584527	KC584195	KC584271	KC584114	KC584395	KC584654
*A. embellisia*	*Embellisia*	CBS 339.71 ^R^	USA	*Allium sativum*	KC584582	KC584230	KC584324	KC584155	KC584449	KC584708
*A. ershadii*	*Pseudoalternaria*	IRAN 3275C	Iran	*Triticum aestivum*	-	MK829647	-	MK829645	-	-
*A. eryngii*	*Panax*	CBS 121339 ^R^	-	*Eryngium* sp.	KC584529	JQ693661	KC584273	AY562416	KC584397	KC584656
*A. euphorbiicola*	*Euphorbiicola*	CBS 119410 ^R^	USA	*Euphorbia pulcherrima*	-	KJ718173	-	KJ718018	KJ718346	KJ718521
*A. euphorbiicola*	*Euphorbiicola*	CBS 133874	USA	*Euphorbia hyssopifolia*	-	KJ718174	-	KJ718019	KJ718347	KJ718522
*A. eureka*	*Eureka*	CBS 193.86 ^T^	Australia	*Medicago rugosa*	KC584589	JN383490	KC584331	JN383471	KC584456	KC584715
*A. gypsophilae*	*Gypsophilae*	CBS 107.41 ^T^	Unknown	*Gypsophila elegans*	KC584533	KC584199	KC584277	KC584118	KC584401	KC584660
*A. helianthiinficiens*	*Helianthiinficientes*	CBS 117370 ^R^	UK	*Helianthus annuus*	KC584534	KC584200	KC584278	KC584119	KC584402	KC584661
*A. helianthiinficiens*	*Helianthiinficientes*	CBS 208.86 ^T^	USA	*Helianthus annuus*	KC584535	JX101649	KC584279	KC584120	KC584403	EU130548
*A. heterospora*	*Ulocladioides*	CBS 123376 ^T^	China	*Lycopersicon esculentum*	KC584621	KC584248	KC584363	KC584176	KC584488	KC584748
*A. heyranica*	*Nimbya*	IRAN 3516C ^T^	Iran	*Carex* sp.	-	MT176114	-	MT187244	MT187270	MT187256
*A. hyacinthi*	*Embellisioides*	CBS 416.71 ^T^	Netherlands	*Hyacinthus orientalis*	KC584590	KC584233	KC584332	KC584158	KC584457	KC584716
*A. indefessa*	*Cheiranthus*	CBS 536.83 ^T^	USA	Soil	KC584591	KC584234	KC584333	KC584159	KC584458	KC584717
*A. infectoria*	*Infectoriae*	CBS 210.86 ^T^	UK	*Triticum aestivum*	KC584536	DQ323697	KC584280	AY278793	KC584404	KC584662
***A. iraniana***	***Iraniana***	**IRAN 5030C ^T^**	**Iran**	***Juncus* sp.**	-	**PV435152**	**PV435160**	**PV443219**	**PV443235**	**PV443227**
***A. iraniana***	***Iraniana***	**FCCUU 1421**	**Iran**	***Juncus* sp.**	-	**PV435153**	**PV435161**	**PV443220**	**PV443236**	**PV443228**
***A. iraniana***	***Iraniana***	**FCCUU 1422**	**Iran**	***Juncus* sp.**	-	**PV435154**	**PV435162**	**PV443221**	**PV443237**	**PV443229**
***A. iraniana***	***Iraniana***	**FCCUU 1423**	**Iran**	***Juncus* sp.**	-	**PV435155**	**PV435163**	**PV443222**	**PV443238**	**PV443230**
*A. japonica*	*Japonicae*	CBS 118390 ^R^	USA	*Brassica chinensis*	KC584537	KC584201	KC584281	KC584121	KC584405	KC584663
*A. junci-acuti*	*Nimbya*	IRAN 3512C ^T^	Iran	*Juncus acutus*	-	MT176113	-	MT187243	MT187269	MT187255
*A. kordkuyana*	*Pseudoalternaria*	IRAN 2764C ^T^	Iran	*Triticum aestivum*	-	MF033843	-	MF033826	-	-
*A. kulundii*	*Soda*	M313 ^T^	Russia	Soil	KJ443087	KJ443262	KJ443132	KJ649618	KJ443176	-
*A. leucanthemi*	*Teretispora*	CBS 422.65 ^R^	USA	*Chrysanthemum maximum*	KC584606	KC584241	KC584348	KC584165	KC584473	KC584733
*A. leucanthemi*	*Teretispora*	CBS 421.65 ^T^	Netherlands	*Chrysanthemum maximum*	KC584605	KC584240	KC584347	KC584164	KC584472	KC584732
*A. limaciformis*	*Phragmosporae*	CBS 481.81 ^T^	UK	Soil	KC584539	KC584203	KC584283	KC584123	KC584407	KC584665
*A. longipes*	*Alternaria*	CBS 540.94 ^R^	USA	*Nicotiana tabacum*	KC584541	AY278835	KC584285	AY278811	KC584409	KC584667
*A. mimicula*	*Brassicicola*	CBS 118696 ^T^	USA	*Lycopersicon esculentum*	KC584543	FJ266477	KC584287	AY562415	KC584411	KC584669
*A. nepalensis*	*Japonicae*	CBS 118700 ^T^	Nepal	*Brassica* sp.	KC584546	KC584207	KC584290	KC584126	KC584414	KC584672
*A. nobilis*	*Gypsophilae*	CBS 116490 ^R^	New Zealand	*Dianthus caryophyllus*	KC584547	KC584208	KC584291	KC584127	KC584415	KC584673
*A. obovoidea*	*Ulocladioides*	CBS 101229	New Zealand	*Cucumis sativus*	KC584618	FJ266487	KC584360	FJ266498	KC584485	KC584745
*A. omanensis*	*Omanenses*	SQUCC 15560	Oman	dead wood	MK878560	MK878563	MK878557	MK880900	MK880894	MK880897
*A. omanensis*	*Omanenses*	SQUCC 13580 ^T^	Oman	dead wood	MK878559	MK878562	MK878556	MK880899	MK880893	MK880896
*A. oregonensis*	*Infectoriae*	CBS 542.94 ^T^	USA	*Triticum aestivum*	KC584548	FJ266478	KC584292	FJ266491	KC584416	KC584674
*A. oudemansii*	*Ulocladium*	CBS 114.07 ^T^	-	-	KC584619	FJ266488	KC584361	KC584175	KC584486	KC584746
*A. panax*	*Panax*	CBS 482.81 ^R^	USA	*Aralia racemosa*	KC584549	KC584209	KC584293	KC584128	KC584417	KC584675
*A. papavericola*	*Crivellia*	CBS 116606 ^T^	USA	*Papaver somniferum*	KC584579	FJ357310	KC584321	FJ357298	KC584446	KC584705
*A. penicillata*	*Crivellia*	CBS 116608 ^T^	Austria	*Papaver rhoeas*	KC584572	FJ357311	KC584316	FJ357299	KC584440	KC584698
*A. penicillata*	*Crivellia*	CBS 116607 ^T^	Austria	*Papaver rhoeas*	KC584580	KC584229	KC584322	KC584153	KC584447	KC584706
*A. perpunctulata*	*Alternantherae*	CBS 115267 ^T^	USA	*Alternanthera philoxeroides*	KC584550	KC584210	KC584294	KC584129	KC584418	KC584676
*A. petroselini*	*Radicina*	CBS 112.41 ^T^	-	*Petroselinum sativum*	KC584551	KC584211	KC584295	KC584130	KC584419	KC584677
*A. petuchovskii*	*Soda*	M304 ^T^	Russia	Alkaline soil	KJ443079	KJ443254	KJ443124	KJ649616	KJ443170	-
*A. photistica*	*Panax*	CBS 212.86 ^T^	UK	*Digitalis purpurea*	KC584552	KC584212	KC584296	KC584131	KC584420	KC584678
*A. phragmospora*	*Phragmosporae*	CBS 274.70	Netherlands	Soil	KC584595	JN383493	KC584337	JN383474	KC584462	KC584721
*A. porri*	*Porri*	CBS 116699 ^T^	USA	*Allium cepa*	-	KJ718218	-	KJ718053	KJ718391	KJ718564
*A. proteae*	*Embellisioides*	CBS 475.90 ^T^	Australia	*Protea* sp.	KC584597	AY278842	KC584339	KC584161	KC584464	KC584723
*A. protenta*	*Porri*	CBS 116651 ^R^	USA	*Solanum tuberosum*	KC584562	KC584217	KC584306	KC584139	KC584430	KC584688
*A. pseudorostrata*	*Porri*	CBS 119411 ^T^	USA	*Euphorbia pulcherrima*	KC584554	JN383483	KC584298	AY562406	KC584422	KC584680
*A. radicina*	*Radicina*	CBS 245.67 ^T^	USA	*Daucus carota*	KC584555	KC584213	KC584299	KC584133	KC584423	KC584681
*A. rosae*	*Pseudoalternaria*	CBS 121341 ^T^	New Zealand	*Rosa rubiginosa*	-	JQ693639	-	JQ646279	-	-
*A. scirpicola*	*Nimbya*	CBS 481.90	UK	*Scirpus* sp.	KC584602	KC584237	KC584344	KC584163	KC584469	KC584728
*A. scirpinfestans*	*Nimbya*	EGS 49-185	USA	*Scirpus acutus*	-	JN383499	-	JN383480	-	-
*A. scirpivora*	*Nimbya*	EGS 50-021	USA	*Scirpus acutus*	-	JN383500	-	JN383481	-	-
*A. scirpivora*	*Nimbya*	IRAN 3421C	Iran	*Scirpus acutus*	-	MT176118	-	MT187248	MT187274	MT187260
*A. selini*	*Radicina*	CBS 109382 ^T^	Saudi Arabia	*Petroselinum crispum*	KC584558	AF229455	KC584302	AY278800	KC584426	KC584684
*A. septospora*	*Pseudoulocladium*	CBS 109.38	Italy	Wood	KC584620	FJ266489	KC584362	FJ266500	KC584487	KC584747
*A. shukurtuzii*	*Soda*	M307 ^T^	Russia	Alkaline soil	KJ443082	KJ443257	KJ443127	KJ649620	KJ443172	-
*A. simsimi*	*Dianthicola*	CBS 115265 ^T^	Argentina	*Sesamum indicum*	KC584560	JF780937	KC584304	KC584137	KC584428	KC584686
*A. smyrnii*	*Radicina*	CBS 109380 ^R^	UK	*Smyrnium olusatrum*	KC584561	AF229456	KC584305	KC584138	KC584429	KC584687
*A. soliaridae*	-	CBS 118387 ^T^	USA	Soil	KC584563	KC584218	KC584307	KC584140	KC584431	KC584689
*A. sonchi*	*Sonchi*	CBS 119675 ^R^	Canada	*Sonchus asper*	KC584565	KC584220	KC584309	KC584142	KC584433	KC584691
*A. tellustris*	*Embellisia*	CBS 538.83 ^T^	USA	Soil	KC584598	MH861643	KC584340	AY562419	KC584465	KC584724
*A. thalictrigena*	-	CBS 121712 ^T^	Germany	*Thalictrum* sp.	KC584568	EU040211	KC584312	KC584144	KC584436	KC584694
*A. triglochinicola*	*Eureka*	CBS 119676 ^T^	Australia	*Triglochin procera*	KC584569	KC584222	KC584313	KC584145	KC584437	KC584695
*A. tumida*	*Embellisioides*	CBS 539.83 ^T^	Australia	*Triticum aestivum*	KC584599	FJ266481	KC584341	FJ266493	KC584466	KC584725
*A. vaccariicola*	*Gypsophilae*	CBS 118714 ^T^	USA	*Vaccaria hispanica*	KC584571	KC584224	KC584315	KC584147	KC584439	KC584697
*Alternariaster bidentis*	-	CBS 134021 ^T^	Brazil	*Bidens sulphurea*	-	KC609333	KC609341	KC609325	KC609347	-
*Alternariaster helianthi*	-	CBS 119672 ^R^	USA	*Helianthus* sp.	KC584626	KC609337	KC584368	KC609329	KC584493	-
*Amarenomyces ammophilae*	-	CBS 114595	Sweden	*Ammophila arenaria*	GU296185	KF766146	GU301859	-	GU371724	-
*Ascochyta pisi*	-	CBS 126.54	Netherlands	*Pisum sativum*	EU754038	-	DQ678070	-	DQ677967	-
*Bipolaris maydis*	-	CBS 137271 ^T^	USA	*Zea mays*	-	AF071325	KM243280	KM034846	-	-
*Bipolaris oryzae*	-	CBS 157.50	Indonesia	*Oryza sativa*	-	HF934931	HF934870	HG779090	HF934833	-
*Bipolaris sorokiniana*	-	CBS 480.74	South Africa	*Tribulus terrestris*	-	KJ909771	KM243282	KM034827	-	-
*Boeremia exigua*	-	CBS 431.74	Netherlands	*Solanum tuberosum*	EU754084	FJ427001	EU754183	-	GU371780	-
*Calophoma complanata*	-	CBS 268.92	Netherlands	*Anglica sylvestris*	EU754081	FJ515608	EU754180	-	GU371778	-
*Chaetosphaeronema hispidulum*	-	CBS 216.75	Germany	*Anthyllis vulneraria*	EU754045	KF251148	EU754144	-	GU371777	-
*Cicatricea salina*	-	CBS 302.84 ^T^	North Sea, Skagerrak	*Cancer pagurus*	KC584583	JN383486	KC584325	JN383467	KC584450	KC584709
*Cnidariophoma eilatica*	-	CPC 44117 ^T^	Israel	*Stylophora pistillata*	-	OQ628480	OQ629062	-	OQ627943	-
*Cochliobolus heterostrophus*	-	CBS 134.39	–	*Zea mays*	AY544727	DQ491489	AY544645	-	DQ247790	-
*Comoclathris compressa*	-	CBS 156.53	USA	*Castilleja miniata*	KC584630	-	KC584372	-	KC584497	-
*Comoclathris incompta*	-	CBS 467.76	Greece	*Olea europaea*	GU238220	-	GU238087	-	KC584504	-
*Comoclathris typhicola*	-	CBS 132.69	Netherlands	*Typha angustifolia*	JF740105	-	JF740325	-	KC584505	-
*Curvularia affinis*	-	CBS 154.34 ^T^	Java	Manihot utilissima	-	HG778981	HG779028	HG779126	HG779159	-
*Curvularia hawaiiensis*	-	BRIP 11987 ^T^	USA	*Oryza sativa*	-	KJ415547	KJ415502	KJ415399	-	-
*Curvularia lunata*	-	CBS 730.96 ^T^	USA	Human lung biopsy	-	JX256429	JX256396	JX276441	HF934813	-
*Decorospora gaudefroyi*	-	CBS 332.63	France	Unknown	AF394542	MH858305	MH869915	-	-	-
*Decorospora gaudefroyi*	-	CBS 250.60	UK	Unknown	-	MH857974	MH869526	-	-	-
*Dichotomophthora lutea*	-	CBS 145.57 ^T^	Unknown	Unknown	-	MH857676	NG069497	LT990663	LT990634	-
*Dichotomophthora portulacae*	-	CBS 174.35 ^T^	Unknown	Unknown	-	NR158421	MH867137	LT990668	LT990638	-
*Didymella glomerata*	-	CBS 528.66	Netherlands	*Chrysanthemum* sp.	EU754085	FJ427013	EU754184	-	GU371781	-
*Didymella maydis*	-	CBS 588.69 ^T^	USA	*Zea mays*	EU754093	FJ427086	EU754192	-	GU371782	-
*Exserohilum corniculatum*	-	BRIP 11426 ^T^	Australia	*Oryza sativa*	-	LT837453	LT883391	LT883533	LT852480	-
*Exserohilum khartoumensis*	-	IMI 249194 ^IsoT^	Sudan	*Sorghum bicolor var. mayo*	-	LT837461	LT715619	LT715888	LT852490	-
*Exserohilum turcicum*	-	CBS 690.71 ^ET^	Germany	*Zea mays*	-	LT837487	LT883415	LT882581	-	-
*Exserohilum turcicum*	-	CBS 387.58	USA	*Zea mays*	-	MH857820	LT883412	LT883554	LT852514	-
*Halojulella avicenniae*	-	BCC 18422	Thailand	Mangrove wood	GU371831	-	GU371823	-	GU371787	-
*Heterosporicola chenopodii*	-	CBS 115.96	Netherlands	*Chenopodium album*	EU754089	JF740227	EU754188	-	GU371775	-
*Johnalcornia aberrans*	-	BRIP 16281 ^T^	Australia	*Eragrostis parviflora*	-	KJ415522	KJ415475	KJ415424	-	-
*Leptosphaeria maculans*	-	DAOM 229267	France	*Brassica* sp.	DQ470993	KT225526	DQ470946	-	DQ470894	-
*Leptosphaerulina australis*	-	CBS 317.83	Indonesia	*Eugenia aromatica*	GU296160	GU237829	GU301830	-	GU371790	-
*Loratospora aestuarii*	-	JK 5535B	USA	*Juncus roemerianus*	GU296168	MH863024	GU301838	-	GU371760	-
*Neocamarosporium betae*	-	CBS 109410	Netherlands	*Beta vulgaris*	EU754079	KY940790	EU754178	-	GU371774	-
*Neocamarosporium calvescens*	-	CBS 246.79	Germany	*Atriplex hastata*	EU754032	KY940774	EU754131	-	KC584500	-
*Neocamarosporium goegapense*	-	CPC 23676 ^T^	South Africa	*Mesembryanthemum* sp.	-	KJ869163	KJ869220	-	-	-
*Neophaeosphaeria filamentosa*	-	CBS 102202	Mexico	*Yucca rostrata*	GQ387516	JF740259	GQ387577	-	GU371773	-
*Neostemphylium polymorphum*	-	FMR 17886 ^T^	Spain	Fluvial sediment	-	OU195609	OU195892	OU195960	OU196009	ON368192
*Neostemphylium polymorphum*	-	FMR 17889	Spain	Fluvial sediment	-	OU195610	OU195914	OU195977	OU196957	ON368193
*Ophiosphaerella herpotricha*	-	CBS 620.86	Switzerland	*Bromus erectus*	DQ678010	-	DQ678062	-	DQ677958	-
*Paradendryphiella arenariae*	-	CBS 181.58 ^T^	France	Coastal sand	KC793336	KF156010	KC793338	-	DQ470924	-
*Paradendryphiella salina*	-	CBS 142.60	UK	*Spartina* sp.	KC793337	DQ411540	KC793339	-	KC793340	-
*Paraleptosphaeria dryadis*	-	CBS 643.86	Switzerland	*Dryas octopetala*	KC584632	JF740213	GU301828	-	GU371733	-
*Phaeosphaeria avenaria*	-	DAOM 226215	Canada	*Avena sativa*	AY544725	-	AY544684	-	DQ677941	-
*Phaeosphaeria eustoma*	-	CBS 573.86	Switzerland	*Dactylis glomerata*	DQ678011	-	DQ678063	-	DQ677959	-
*Phoma herbarum*	-	CBS 276.37	Sweden	Wood pulp	DQ678014	-	DQ678066	-	DQ677962	-
*Porocercospora seminalis*	-	CBS 134907	USA	*Bouteloua dactyloides*	-	HF934941	HF934862	-	HF934843	-
*Porocercospora seminalis*	-	CPC 213.49	USA	*Bouteloua dactyloides*	-	HF934945	HF934861	-	HF934845	-
*Pyrenophora avenicola*	-	CBS 307.84 ^T^	Sweden	*Avena* sp.	-	MK539972	MK540042	MK540180	-	-
*Pyrenophora phaeocomes*	-	DAOM 222769	Switzerland	*Calamagrostis villosa*	DQ499595	JN943649	DQ499596	-	DQ497614	-
*Scleromyces submersus*	-	FMR 18289 ^T^	Spain	Fluvial sediment	-	OU195893	OU195959	OU196008	OU197244	OU196982
*Setomelanomma holmii*	-	CBS 110217	USA	*Picea pungens*	GU296196	KT389542	GU301871	-	GU371800	-
*Stemphylium botryosum*	-	CBS 714.68 ^T^	Canada	*Medicago sativa*	KC584603	KC584238	KC584345	AF443881	AF107804	KC584729
*Stemphylium vesicarium*	-	CBS 191.86 ^T^	India	*Medicago sativa*	GU238232	KC584239	GU238160	AF443884	KC584471	KC584731
*Tamaricicola muriformis*	-	MFLUCC 150488	Italy	*Tamarix* sp.	KU870909	KU752187	KU561879	-	KU820870	-
*Tamaricicola muriformis*	-	MFLUCC 150489	Italy	*Tamarix* sp.	KU870910	KU752188	KU729857	-	-	-
*Typhicola typharum*	-	CBS 145043 ^NT^	Germany	Leaf of *Typha* sp.	-	MK442590	MK442530	-	MK442666	-

**Table 3:** Phylogenetic information of individual and combined sequence datasets used in phylogenetic analyses.

Analysis	Region/Gene	Parameter								
		Number of Taxa	Total Characters	Constant Sites	Variable Sites	Parsimony Informative Sites	Parsimony Uninformative Sites	AIC Substitution Model *	Lset nst, Rates	−lnL
First analysis (suborder *Pleosporineae*)	SSU	122	1373	1194	179	69	110	GTR+I+G	6, invgamma	3432.2908
	ITS	158	437	266	171	143	28	GTR+I+G	6, invgamma	5161.6314
	LSU	150	869	686	183	113	70	GTR+I+G	6, invgamma	3733.1211
	*GAPDH*	130	534	302	232	207	25	GTR+I+G	6, invgamma	8174.018
	*RPB2*	150	837	359	478	419	59	GTR+I+G	6, invgamma	21800.52
	Combined	167	4050	2807	1243	951	292	GTR+I+G	6, invgamma	44380.022
Second analysis (All *Alternaria* sections)	SSU	89	1022	982	40	27	13	GTR+I	6, propinv	1813.347845
	ITS	110	457	336	121	95	26	SYM+I+G	6, invgamma	2892.446526
	LSU	93	851	798	53	36	17	GTR+I+G	6, invgamma	1734.095321
	*GAPDH*	110	557	334	223	195	28	GTR+I+G	6, invgamma	6256.718561
	*RPB2*	103	799	500	299	285	14	GTR+I+G	6, invgamma	9217.307515
	*TEF1*	100	317	155	162	139	23	GTR+I+G	6, invgamma	3802.639587
	Combined	110	4003	3105	898	777	121	SYM+I+G	6, invgamma	27448.64266

* Akaike Information Criterion Substitution models implemented in Bayesian Inference.

## Data Availability

All of the data supporting the findings of this study are available in the main text.
